# Highly Pathogenic Fowlpox Virus in Cutaneously Infected Chickens, China

**DOI:** 10.3201/eid2007.131118

**Published:** 2014-07

**Authors:** Kui Zhao, Wenqi He, Shengnan Xie, Deguang Song, Huijun Lu, Wei Pan, Ping Zhou, Wenfeng Liu, Rongguang Lu, Jiyong Zhou, Feng Gao

**Affiliations:** Jilin University, Changchun, China (K. Zhao, W. He, S. Xie, D. Song, H. Lu, W. Pan, P. Zhou, W. Liu, R. Lu, F. Gao);; Zhejiang University, Hangzhou, China (J. Zhou)

**Keywords:** fowlpox, viruses, chickens, cutaneous infection, highly pathogenic, China

## Abstract

We investigated an acute outbreak of the cutaneous form of fowlpox among chickens in China in November 2009. Using pathologic and virologic methods, we identified a novel type of fowlpox virus that carried an integrated genomic sequence of reticuloendotheliosis virus. This highly pathogenic virus could lead to severe ecologic effects and economic losses.

Fowlpox has been reported worldwide as a mild to severe poultry disease (*1*). Caused by fowlpox virus (FWPV), the disease is primarily found in 2 forms, cutaneous and diphtheritic (*2*). The cutaneous form is usually mild and characterized by multifocal cutaneous lesions on unfeathered areas of the skin. The more severe diphtheritic form is characterized by fibrous necrotic proliferative lesions on the mucous membranes of the respiratory and gastrointestinal tracts and causes more deaths than the cutaneous form, usually resulting from asphyxiation.

In recent years, fowlpox outbreaks in poultry flocks have been gradually increasing because of an emerging novel type of FWPV (*3*–*5*). The pathogenic traits of this virus type are likely enhanced by integrated reticuloendotheliosis virus (REV) sequences of various lengths in the FWPV genome (*6*–*8*). Although this variant FWPV has been found widely (*7*,*9*–*14*), the reported illness and death rates from the cutaneous form of fowlpox in chickens have not reached 100%. We investigated a severe outbreak of cutaneous fowlpox in a poultry flock in northeastern China in which all infected chickens died. The flock had not been vaccinated with an FWPV vaccine.

## The Study

In November 2009, a natural outbreak of the cutaneous form of fowlpox occurred in a poultry flock in Jilin Province in northeastern China (125°35′ E, 43°88′ N). A total of 10,000 brown breeding, 46-day-old chickens (Jilin Zhengda Co., Ltd, Changchun, China) used for egg production were affected. The flock had not received vaccination against FWPV.

**Figure 1:**
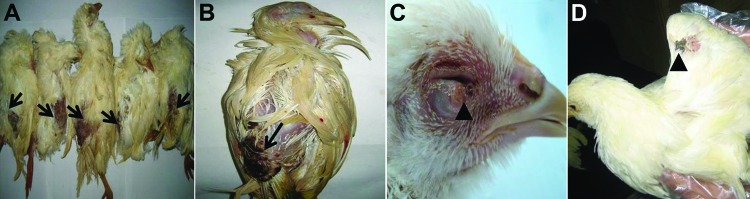
Macropathologic images of fowlpox virus infection in chickens from a commercial flock in northeastern China. A, B) Severe lesions on the skin in unfeathered areas of the backs (arrows). C) Cutaneous exanthema variolosum of the eyelids (arrowhead). D) Skin pock lesions in the wings (arrowhead).

Clinical signs, including listlessness, anorexia, and typical skin pock lesions, were observed in all infected chickens. Lesions types varied in size and type: ulcerated, multifocal, or coalescing proliferative cutaneous exanthema variolosum. The lesions appeared on the skin in unfeathered areas of the backs, the eyelids, and the wings (Figure 1). All of the birds died within 10 days after clinical signs first appeared.

Postmortem examinations were performed for pathol ogic evaluation. Samples submitted for histopathologic examination included skin from the varioliform exanthema areas, trachea, thymus gland, bursa of fabricius, and internal organs. Microscopic examination of skin lesions showed swelling, vacuolation, and characteristic eosinophilic cytoplasmic inclusion bodies in the stratified squamous epithelial cells of the folliculus pili (Technical Appendix Figure 1). No obvious lesions were observed in other organs.

Electron microscopy of the clarified supernatant of the scab specimens collected from the skin of infected chickens showed characteristic FWPV virions, which have an ovoid shape (Technical Appendix Figure 1). We attempted to isolate the virus by injecting the chorioallantoic membranes (CAM) and allantoic cavities of 9-day-old specific pathogen free (SPF) chicken embryos with the scab specimens that were positive for FWPV. White, raised varioles were observed on the CAMs of the embryos 4 days after injection (Technical Appendix Figure 1). Electron microscopy also showed FWPV-shaped virions in the supernatant of the CAMs. After 5 blind passages at 4-day intervals, no other viruses were isolated from the allantoic cavities of the SPF chicken embryos.

We used indirect immunofluorescence and a DF-1 chicken embryo fibroblast cell line to test the ability of the FWPV isolate from the CAMs to invade cells in vitro. Chicken anti-FWPV polyclonal antibody was used as the primary antibody; the secondary antibody was fluorescein isothiocyanate–conjugated goat anti-chicken IgG. Cellular nuclei were stained by using 4′,6-diamidino-2-phenylindole. In some cells, typical bright, DNA-containing poxvirus factories were evident, often coincident with virus antigen–specific green fluorescence, at 3 days postinfection (dpi) (Technical Appendix Figure 1).

Viral genomic DNA was extracted from scab specimens, and PCR amplification was performed immediately by using the specific primers for FWPV P4b gene (P4b Fw1: 5′-GATAGAGGATCGTACATCCA-3′; and P4b Rv1: 5′-CATCTACTCATGACTGGCAA-3′). The size of the product was 1,381 bp (Technical Appendix Figure 2). The amplicons were sequenced, and the obtained P4b gene sequence was submitted to GenBank (accession no. KF875986). We then used the neighbor-joining method in MEGA4 (*15*) to construct a phylogenetic tree on the basis of the nucleotide sequences of P4b gene with corresponding reference sequences (Technical Appendix Figure 3). The resulting tree showed that the FWPV isolate clustered in the same branch with other FWPVs and that the P4b gene shared a close relationship with other FWPVs (99.9%–100%). This result indicates that the P4b genes were highly conserved among FWPV isolates. No nucleic acid sequences of other potentially pathogenic viruses (i.e., avian influenza virus, Newcastle disease virus, Marek’s disease virus, chicken anemia virus, avian leukosis virus J subgroup, infectious bursal disease virus) were detected by using PCR or reverse transcription PCR. These findings indicate that FWPV may have been the causative pathogen in the infected chickens.

**Figure 2:**
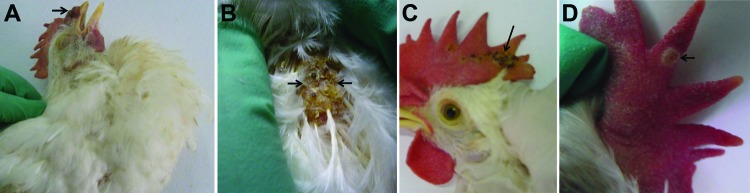
Macropathologic images of fowlpox virus infection in experimentally infected specific pathogen free (SPF) chickens. China. A) Brown variolar crusts on the combs of 18-day-old SPF chickens at 14 days postinfection (dpi). B) Large areas of brown scabs on the backs of 18-day-old SPF chickens at 14 dpi. C) Multifocal to coalescing pock lesions on the combs of 53-day-old SPF chickens at 14 dpi. D) Cutaneous exanthema variolosum on the combs of 145-day-old SPF chickens at 14 dpi.

To investigate the possibility of an integration of an REV gene sequence into the FWPV genome, we designed another 2 sets of primers for the amplification of a partial REV *env* gene and the REV *env*–FWPV open reading frame 203, which contains the entire REV 3′ long terminal repeat. The primer sequences were as follows: REV-env Fw1, 5′-ACCACTCTCGACTCAAGAAA-3′; REV-env Rv1, 5′-CCACACACAAATACATGACCC-3′; REV env-FWPV 203 Fw1, 5′-GAAATCTTACGAGGCTATGTC-3′; and REV env-FWPV 203 Rv1, 5′-TTCAACCACCAGGCTACATAAAGG-3′. Specific products of the expected sizes, 1,089 bp and 1,437 bp, were amplified from the skin lesions (Technical Appendix Figure 2). The results indicated that the FWPV isolate had integrated partial REV sequences.

We further determined the pathogenesis of the FWPV isolate by experimentally infecting 18-day-old, 53-day-old, and 145-day-old SPF chickens. The experimental groups (10 chickens per group) were inoculated by scarification of the wing and skin scarification into the feather folliculus pili by using purified virus containing 200 50% egg infectious doses of the virus. A control group (10 chickens) was injected with 0.2 mL of phosphate-buffered saline. All inoculated chickens had characteristic skin pock lesions develop at 7–14 dpi (Figure 2) and died at 18–25 dpi; illness and death rates were 100%. Scab specimens were collected at 7, 9, 14, and 20 dpi for histologic examination. The chickens in the control group did not show any clinical signs.

The paraffin sections of scab samples from the SPF chickens inoculated with FWPV were positive not only for FWPV, tested by using a chicken anti-FWPV polyclonal antibody, but also for REV, tested by using a monoclonal antibody that specifically recognized the envelope protein of REV in the cytoplasm of stratified squamous epithelial cells of the folliculus pili by immunohistochemical assay (Technical Appendix Figure 1).

## Conclusions

Our investigation of an acute outbreak of the cutaneous form of fowlpox determined that the outbreak was caused by a novel type of FWPV that carried integrated REV genomic sequences. Illness and death rates of up to 100% occurred in this commercial poultry flock in northeast China. Our results show that the novel FWPV we isolated was much more pathogenic than common FWPV strains obtained from other chickens infected with the cutaneous form of fowlpox. This highly pathogenic FWPV variant is a potential threat to chickens and could lead to severe ecologic effects and economic losses. The virulence of this FWPV is probably dependent on the presence of the REV sequences in the FWPV genome, although this conclusion needs experimental confirmation. Because these sequences are also found in less virulent isolates, other determinants may account for this unusual phenotype. Identifying the genomic changes responsible for the increased pathogenicity of this FWPV variant will require considerable effort in sequencing and molecular virology.

Technical Appendix Imaging of lesions and plaques in infected chickens and fowlpox virus, amplification of fowlpox virus genes, and phylogenetic analysis of fowlpox virus isolates.
