# Supplementary material for: Highly Pathogenic Fowlpox Virus in Cutaneously Infected Chickens, China
Source: Emerg Infect Dis. 2014 Jul;20(7):1200–2. doi: 10.3201/eid2007.131118 (PMC4073872; doi:10.3201/eid2007.131118)
Supplement: Technical Appendix — Imaging of lesions and plaques in infected chickens and fowlpox virus, amplification of fowlpox virus genes, and phylogenetic analysis of fowlpox virus isolates. [file 13-1118-Techapp-s1.pdf]

Article DOI: http://dx.doi.org/10.3201/eid2007.131118

Highly Pathogenic Fowlpox Virus in Cutaneously Infected Chickens, China

Technical Appendix

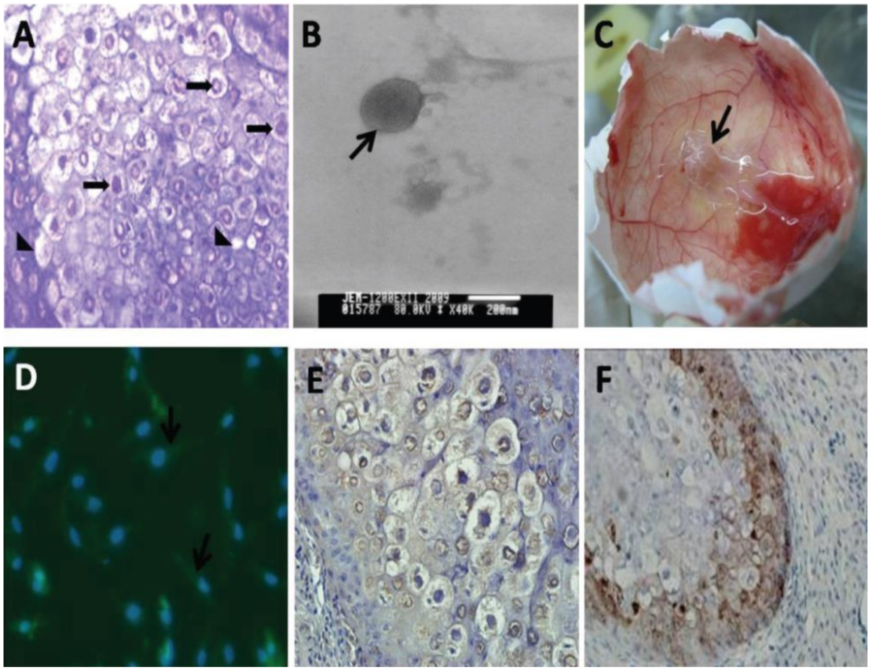

Technical Appendix Figure 1. A) Microscopy of skin lesions collected from fowlpox virus (FWPV)–infected chickens showing ballooning degeneration (arrowheads) and characteristic eosinophilic cytoplasmic inclusion bodies (black arrows) in the stratified squamous epithelial cells of the folliculus pili (hematoxylin and eosin stain;original magnification ×400). B) The characteristic FWPV virus virions with an ovoid-shaped appearance were observed in transmission electron microscopy. The virions are ≈340 nm in length and 280 nm in width. Scale bar = 200 nm. C) The white elevated plaques were observed on the chorioallantoic membrane of inoculated specific pathogen free chicken embryos 4 days postinoculation. D) The cellular nuclei were stained blue by 4',6-diamidino-2-phenylindole. In some cells (arrows), typical bright, perinuclear, DNA-containing poxvirus factories were evident, often coincident with virus antigen-specific green fluorescence. E) FWPV antigens were identified using immunostaining of the epithelium tissues collected from the scabs of chickens infected with the FWPV field isolate. Tissues stained brown show positive immunoreactivity. Nuclei are stained blue. F) Reticuloendotheliosis virus antigens were identified using immunohistochemistry technique in the epithelium tissues collected from the scabs of chickens infected with the FWPV field isolate. Tissues stained brown show positive immunoreactivity. Nuclei are stained blue.

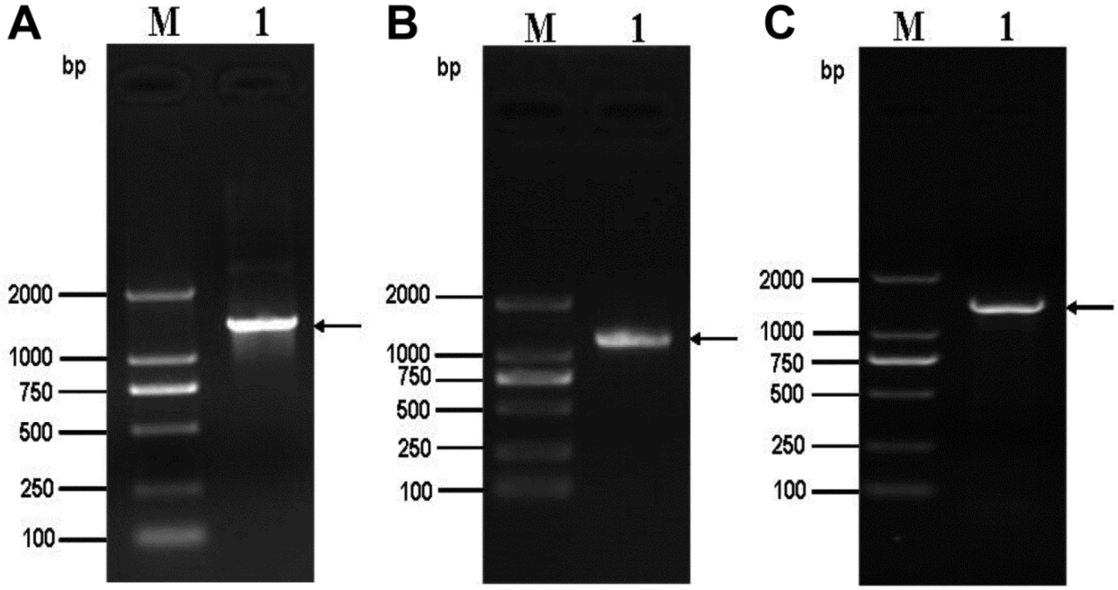

Technical Appendix Figure 2**.** Amplification of the fowlpox virus (FWPV) P4b gene, reticuloendotheliosis virus (REV) env gene, and REV env-FWPV ORF203 by PCR. A) Lane 1: PCR product (1381 bp) of FWPV P4b gene (arrow); M: DL 2000 DNA Marker (bp). B) Lane 1: PCR product (1089 bp) of partial REV env gene (arrow); M: DL 2000 DNA Marker (bp). C) Lane 1: PCR product (1437 bp) containing the partial REV env gene and complete REV 3'-LTR (arrow); M: DL 2000 DNA Marker (bp).

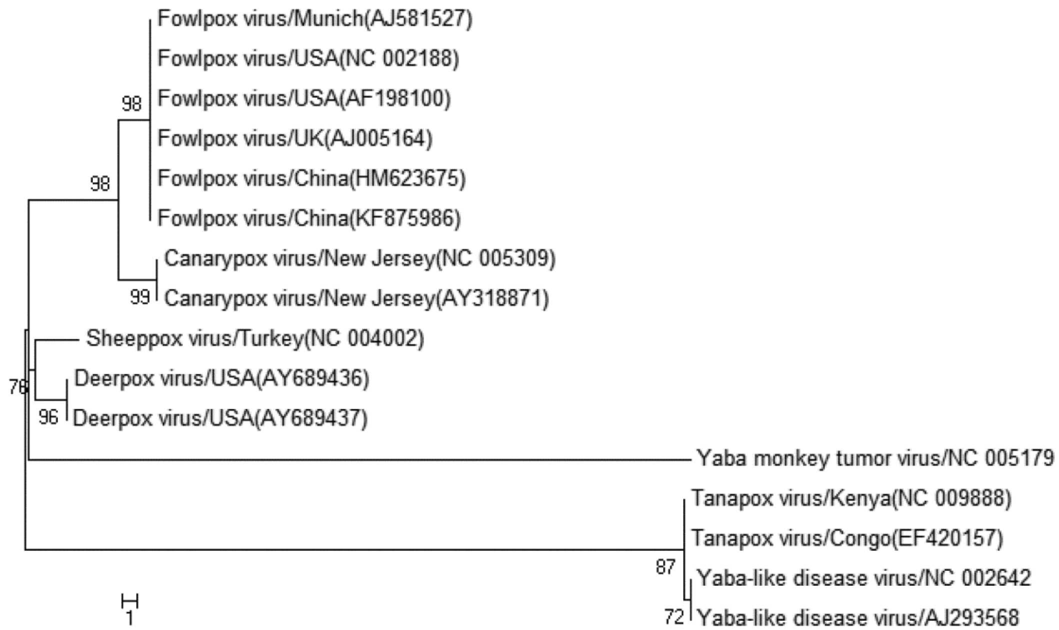

Technical Appendix Figure 3. Phylogenetic analysis of different fowlpox virus (FWPV) isolates based on nucleotide sequences of FWPV P4b gene.
